# α-synuclein transfer through tunneling nanotubes occurs in SH-SY5Y cells and primary brain pericytes from Parkinson’s disease patients

**DOI:** 10.1038/srep42984

**Published:** 2017-02-23

**Authors:** Birger Victor Dieriks, Thomas I-H. Park, Chantelle Fourie, Richard L. M. Faull, Mike Dragunow, Maurice A. Curtis

**Affiliations:** 1 Department of Anatomy and Medical Imaging, Faculty of Medical and Health Science, University of Auckland, Private Bag 92019, Auckland, New Zealand; 2 Centre for Brain Research, Faculty of Medical and Health Science, University of Auckland, Private Bag 92019, Auckland, New Zealand; 3 Department of Pharmacology, Faculty of Medical and Health Science, University of Auckland, Private Bag 92019, Auckland, New Zealand; 4 Department of Physiology, Faculty of Medical and Health Science, University of Auckland, Private Bag 92019, Auckland, New Zealand

## Abstract

Parkinson’s disease (PD) is characterized by the presence of inclusions known as Lewy bodies, which mainly consist of α-synuclein (α-syn) aggregates. There is growing evidence that α-syn self-propagates in non-neuronal cells, thereby contributing to the progression and spread of PD pathology in the brain. Tunneling nanotubes (TNTs) are long, thin, F-actin-based membranous channels that connect cells and have been proposed to act as conduits for α-syn transfer between cells. SH-SY5Y cells and primary human brain pericytes, derived from postmortem PD brains, frequently form TNTs that allow α-syn transfer and long-distance electrical coupling between cells. Pericytes *in situ* contain α-syn precipitates like those seen in neurons. Exchange through TNTs was rapid, but dependent on the size of the protein. Proteins were able to spread throughout a network of cells connected by TNTs. Transfer through TNTs was not restricted to α-syn; fluorescent control proteins and labeled membrane were also exchanged through TNTs. Most importantly the formation of TNTs and transfer continued during mitosis. Together, our results provide a detailed description of TNTs in SH-SY5Y cells and human brain PD pericytes, demonstrating their role in α-syn transfer and further emphasize the importance that non-neuronal cells, such as pericytes play in disease progression.

Parkinson’s disease (PD) is the second most common neurodegenerative disease after Alzheimer’s disease^1^. Pathologically, PD is characterized by the presence of intracellular inclusions called Lewy bodies. The main protein component of Lewy bodies is α-synuclein (α-syn), a synaptic protein that has a conformational plasticity allowing various structural conformations. In PD, α-syn misfolds and subsequently forms aggregates^2^. Landmark studies designed to investigate early origin and progressive spread of α-syn throughout the human brain demonstrated that the olfactory bulb and locus coeruleus are the regions that are affected first in the body. It is only in later stages of the disease that Lewy bodies are seen in the neocortex and substantia nigra, and this is also when the main motor symptoms of PD become apparent^3^. Therefore, it appears that α-syn precipitates spread from early affected brain regions, to more central areas of the brain. Other studies support this ‘α-syn spread’ theory. Intrastriatal grafts from healthy embryonic dopaminergic neurons, given to PD patients, contained α-syn-positive Lewy bodies when the brain was autopsied more than 10 years later^4^,^5^. α-syn is not restricted to the central nervous system and is able to cross the blood-brain barrier in both directions^6^, with α-syn and its phosphorylated form being present in human blood plasma^7^. Pericytes are uniquely positioned within the neurovascular unit between endothelial cells of brain capillaries, astrocytes and neurons^8^. Pericytes regulate the key neurovascular functions including blood–brain barrier formation and maintenance. In Alzheimer’s disease pericyte deficiency directly leads to the development of tau pathology and an early neuronal loss that is normally absent in Aβ-precursor protein transgenic mice^9^. Because of this transport and a dysfunctional blood-brain barrier influencing pathogenesis and progression in PD, it is important to look at the role that pericytes play in the spread of α-syn in PD^8^.

Tunneling nanotubes (TNTs) have been shown to act as a conduit for α-syn transfer in mouse neuron-like CAD cells^10^, but there is no published evidence of this in human non-neuronal cells such as pericytes. TNTs are long, thin, F-actin-based membranous channels that connect cells and allow transfer of materials^11^,^12^. TNT diameter typically ranges from 50–200 nm and their length can vary dramatically and reach up to several cell diameters^12^. TNTs are very dynamic structures and can connect cells for several seconds up to multiple hours. Currently, there are two proposed models for TNT formation. The actin-driven protrusion mechanism involves one or two protrusive events that connect the membrane of the two cells. The cell-dislodgement mechanism involves two cells in close contact that allow their membranes to fuse, and as the cells migrate away from each other, TNTs are formed, composed of membrane originating from either one or both cells^13^,^14^. There are no known specific TNT markers, making them hard to study. To avoid this confusion with similar-looking structures, it is pivotal to clearly define TNTs. The following TNT definition was recently proposed: TNTs contain actin, attach two cells and are not attached to the substrate^11^. In addition, the transfer of a signal or cargo needs to be added as an essential distinguishing characteristic of TNTs. Only this criterion allows for the differentiation of TNTs from other similar structures that function in movement and adherence as opposed to communication.

In this study, we determined that SH-SY5Y cells and human primary brain pericytes use TNTs as a mechanism for intercellular α-syn transfer and show that TNTs provide for the transport of α-syn pathology. Our results suggest the possibility that TNTs assist in the spread of α-syn throughout the brain and emphasize the role that non-neuronal cells such as pericytes play in α-syn progression in PD.

## Results

### α-synuclein transfers through tunneling nanotubes in SH-SY5Y cells

**Figure 1:**
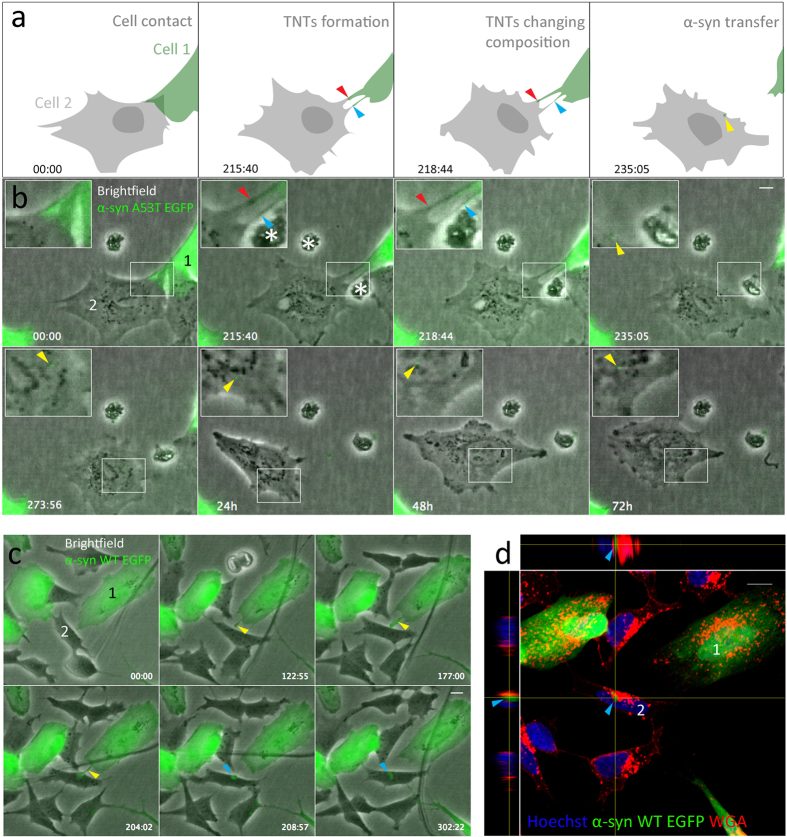
Transfer of α-synuclein (α-syn) in SH-SY5Y cells through tunneling nanotubes (TNTs). (**a**) Representation (as shown in B) of α-syn exchange through TNTs formed by cell dislodgement. The two TNTs are made up of membrane from cell 1 and cell 2 and changes during the time lapse (red and blue arrows). After the TNT retracts an α-syn particle (yellow arrow) is seen in cell 2. (**b**) α-syn A53T-EGFP exchange in SH-SY5Y cells. Cell 1 moves away from cell 2, thereby forming two TNTs. After 235 min the top TNT breaks off and an α-syn-A53T-EGFP particle (yellow arrow) remains in the non-transfected cell 2. This particle remains visible in the accepting cell until recording finishes (72 h). (**c**) α-syn-WT-EGFP exchange. TNT is visible between 122–204 min (yellow arrows). The transferred particle is visible from 208 min until the end of recording (302 min, blue arrows). (**d)** Subsequent confocal recording, with orthogonal views, of the accepting cell 2 (from B) showing internalized α-syn-EGFP particle (blue arrows). Scale bars represent 10 μm. *Indicates cell debris.

To understand whether α-syn can be intercellularly transferred, we set up co-cultures of SH-SY5Y cells stably expressing α-syn-WT-EGFP, α-syn-A53T-EGFP, α-syn-WT-mCherry, mCherry (control), and SH-SY5Y cells that were not transfected. SH-SY5Y cells frequently formed what appeared to be TNTs and could transport fluorescent α-syn between SH-SY5Y cells. SH-SY5Y cells formed TNTs through both cell dislodgment and protrusion in our experiments. Independent of the mechanism, two neighboring cells would make contact through TNTs, the TNT retracts and a transferred particle is then observed in the accepting cell. The particle remained visible for a variable amount of time. This is schematically represented (Fig. 1A) based on the results in Fig. 1B.

SH-SY5Y cells overexpressing α-syn-A53T-EGFP or α-syn-WT-EGFP transferred α-syn through TNTs. No obvious differences were seen between the different α-syn constructs and control proteins (mcherry only). Because of the large variation observed in TNTs, we included various examples of TNT mediated transfer. SH-SY5Y cells formed TNTs through cell dislodgment exchanging α-syn-A53T-EGFP. In our experiments the exchanged α-syn-A53T-EGFP particles remained visible for 72 hours (Fig. 1B). However, most TNT mediated transfer resulted in a transferred α-syn-A53T-EGFP particle that gradually disappeared after several hours (Movie S1). As the accepting cell subsequently goes through mitosis, no α-syn-A53T-EGFP particles were detected in the daughter cells; no immediate toxic effects were apparent after TNT mediated transfer. After mitosis, the daughter cells continued forming TNTs.

SH-SY5Y cells transferred α-syn-WT-EGFP through TNTs in the same manner as α-syn-A53T-EGFP (Fig. 1C). A TNT is formed through protrusion and an α-syn-WT-EGFP particle is transferred from cell 1 to the accepting cell 2. 310 min after the start of the recording, the cells were fixed and processed for confocal imaging. Wheat germ agglutinin (WGA) was used to label all the membranes, and the confocal image and corresponding orthogonal views of cell 2 demonstrate that the α-syn-WT-EGFP is internalized (Fig. 1D). Overexpressed mCherry protein was also transferred through TNTs (Movie S2). A TNT formed by cell dislodgement during mitosis (1062 min) enabled a transfer of an mCherry particle and remained visible for over 6 hours. These observations revealed that SH-SY5Y cells formed additional TNTs with interacting cells during mitosis.

### Transfer of membrane

To further elucidate the exact mechanism of TNT transfer, we used a membrane-RFP-tagged BacMam virus to transfect cells and visualize their membranes. This technique allowed us to observe the composition of TNTs more accurately and see more TNTs, including thinner TNTs that are not visible with brightfield microscopy.

**Figure 2:**
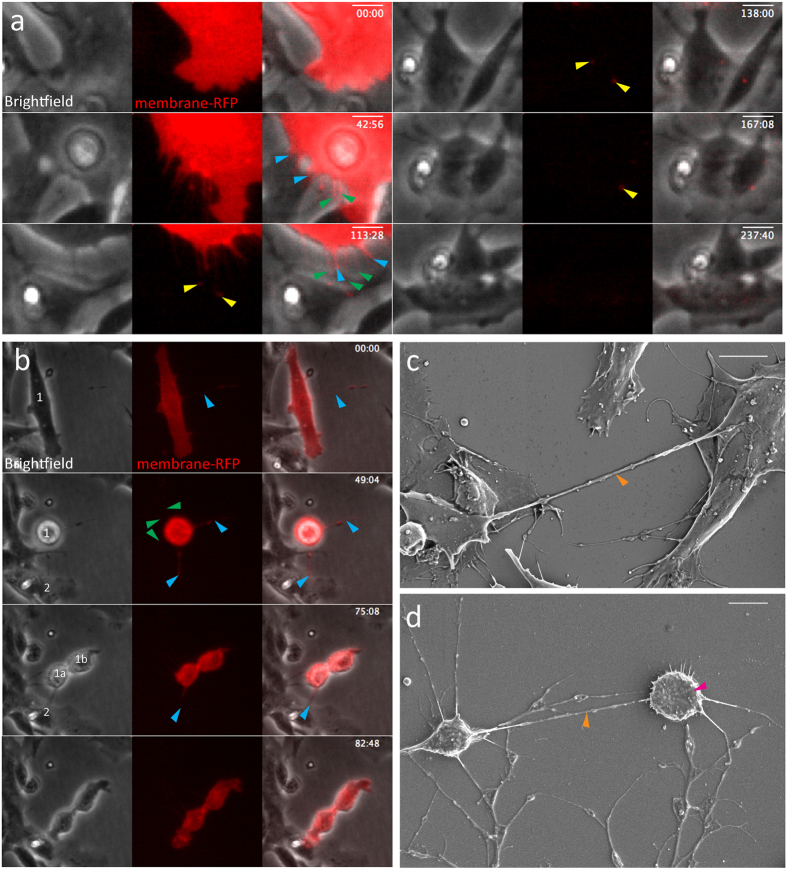
Exchange of membrane-RFP in SH-SY5Y cells through tunneling nanotubes (TNTs). Some TNTs are visible with brightfield and membrane-RFP (blue arrows), whereas thinner TNTs are only seen with membrane-RFP (green arrows). (**a**) Formation of TNTs of variable thickness in SH-SY5Y cells. Membrane is exchanged with the other SH-SY5Y cell (yellow arrows), but gradually disappears throughout the recording. (**b**) Increased formation of TNTs in SH-SY5Y cell 1 during mitosis (blue and green arrows) with the substrate and the neighboring cells. No membrane exchange is observed during this mitosis (**c**) SEM image of SH-SY5Y cells connected by a TNT (orange arrow). (**d**) SEM image of a mitotic SH-SY5Y cell (pink arrow) connected by a TNT (orange arrow). Scale bars represent 10 μm.

Similar to what we observed with overexpressed α-syn and control plasmids, we saw the exchange of membrane-RFP through TNTs. Some TNTs were visible in brightfield and membrane-RFP (blue arrows), whereas thinner TNTs were only seen with membrane-RFP (green arrows; Fig. 2). These thin TNTs were formed in a similar way to the TNTs that were visible with brightfield, and some were formed by thicker TNTs that elongated until they were no longer visible with brightfield microscopy. In this study, after 113 min, two membrane-RFP spots were visible and transferred through TNTs into neighboring cells (yellow arrows). As the time-lapse recording continued, these spots gradually disappear. At the end of the recording, no RFP was visible in the accepting cells (237 min; Fig. 2A). The transfer of membrane was observed more frequently than α-syn TNT-mediated exchange, and with membrane-RFP as a marker, more TNTs could be seen compared to only using brightfield.

During mitosis, an increase of TNTs was observed. The TNTs formed during mitosis did not always result in observable membrane exchange (Fig. 2B). In this figure, two daughter cells are formed during mitosis (49 min). The bottom TNT (blue arrow) did not detach and no membrane exchange was observed. Instead, it is likely that the TNT functioned as a guide for the daughter cell, cell 1a, to attach to cell 2 (Fig. 2B).

Using scanning electron microscopy (SEM) TNTs were observed between neighboring SH-SY5Y cells (Fig. 2C) and cells going through mitosis (Fig. 2D); the images display typical examples of TNTs connecting two cells. Unlike other cellular processes, TNTs generally form in a straight line between SH-SY5Y cells *in vitro*, and do not appear to attach to the substrate. We did observe examples of TNTs *in vitro* that connected two cells that had a third cell in between them, so that the TNT had to go around the third cell, but these were not observed using SEM. TNTs formed during mitosis (Fig. 2D) have a similar appearance to TNTs that are formed in non-mitotic cells (Fig. 2C).

### α-syn and membrane transfer through tunneling nanotubes in primary human brain pericytes from PD patients

**Figure 3:**
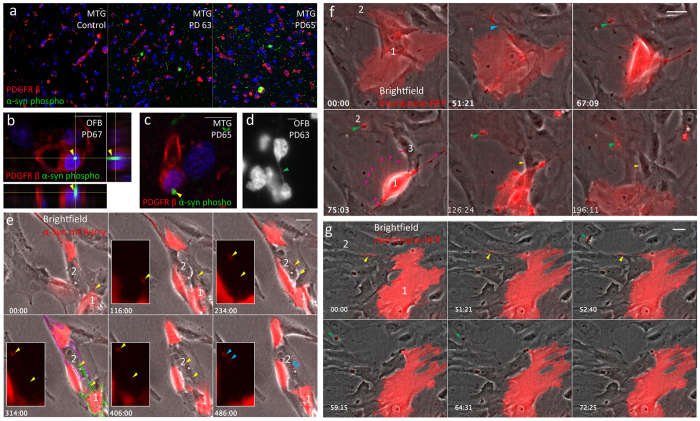
Expression of α-synuclein (α-syn) in human pericytes stained with PDGFRβ and transfer through tunneling nanotubes. (**a**) Distribution and localisation of α-syn precipitates and PDGFRβ, a pericyte-specific marker in human control and Parkinson’s disease middle temporal gyrus (MTG). (**b**) Confocal recording with orthogonal views of a pericyte cell with an internalized α-syn precipitate (yellow arrows) in the olfactory bulb (OFB). (**c**) Single confocal plane of a pericyte cell, adjacent to an α-syn precipitate in MTG. (**d**) *In vivo* TNT-like nanotubes (green arrow) in OFB labeled with Hoechst. (**e**) Transfer of α-syn-mCherry through tunneling nanotubes (TNTs) in pericytes *in vitro*. Two pericytes move away from each other thereby forming a TNT (yellow arrows) between cells 1 (traced in green) and 2 (traced in pink). After 406 min the TNT is no longer visible and two α-syn mCherry particles (blue arrows) remain in the non-transfected cell 2 until the end of the recording (486 minutes). (**f**) Exchange of membrane-RFP between pericytes before and during mitosis. Before mitosis two pericytes (cells 1 and 2) move away from each other, forming a TNT (blue arrow). Membrane (green arrow) is transferred to cell 2 and remains there throughout the recording. At 75 min cell 1 goes through mitosis and forms several connections (pink arrows) with the substrate and neighboring cells. Most of these connection/TNTs are not visible with brightfield. One TNT allows exchange of membrane (yellow arrow) with cell 3 and remains there throughout the recording (196 min). (**g**) Exchange of membrane-RFP between pericytes. After 51 min one of the pericytes (cell 2) moves away, forming a TNT (yellow arrow) between cells 1 and 2. The TNT breaks off and membrane-RFP (green arrow) is exchanged with cell 2. The membrane-RFP quickly dissipates and is no longer visible 20 min after transfer (at 72 min). Scale bars represent 10 μm (**a–d**) and 30 μm (**e–g**).

A high load of α-syn phospho precipitates were found in both PD MTG cases (Fig. 3A). Because the olfactory bulb is the first area where α-syn precipitates accumulate within the human body we also studied the olfactory bulbs. In both MTG and olfactory bulb, α-syn phospho precipitates were located in close proximity to pericytes (labeled with PDGFRβ^15^), and some pericytes in the olfactory bulb had internalized α-syn phospho precipitates, as observed with confocal microscopy (Fig. 3B–C). We screened the olfactory bulbs and MTG regions of PD63, PD65, PD67 for the presence of TNT-like structures. We found examples of short TNT-like processes similar to TNTs in the olfactory bulb of PD63 (Fig. 3D green arrow). Because of the abundant expression of α-syn aggregates in the MTG, the presence of internalized α-syn precipitates and TNT-like structures in olfactory bulb pericytes, MTG pericytes were deemed relevant for this PD study.

We investigated if cultured human pericytes were capable of exchanging α-syn through TNTs. These cultures were slower to grow than SH-SY5Y cells and entered senescence after passage 10. Therefore, we chose to perform transient transfection to overexpress fluorescent α-syn. Pericyte cells are highly variable in shape; the outlines of cell 1-2 is depicted here as an example (Fig. 3E). The transferred α-syn mCherry particles remained visible in the receiving cell until the end of the recording (blue arrows, 486 min). Pericytes transferred α-syn through TNTs in a similar manner as SH-SY5Y cells.

The transfer of pericyte membranes labeled with membrane-RFP also occurred frequently via TNTs before and during mitosis. In contrast to our observations with SH-SY5Y cells, the vast majority of TNT formation in pericytes occurred through cell dislodgement (Fig. 3E–G). TNTs became visible when the cells were moving away from each other. As in SH-SY5Y cells, these primary pericytes also increased the number of TNTs with neighboring cells during mitosis. Ten TNTs (pink arrows) were counted during mitosis of cell 2 (Fig. 3F, bottom panels), where only 2 TNTs were counted before mitosis. For the vast majority of these TNTs, no transfer of membrane-RFP could be observed. The sole TNT that resulted in membrane-RFP transfer was connecting cells 1 and 3. After mitosis the TNT was no longer visible, but a small membrane-RFP particle was observed in cell 3 (yellow arrow). Together with the already transferred membrane particle in cell 2 (green arrow), this particle in cell 3 remained visible until the end of the recording (196 min). In some cases, TNT transfer of membrane-RFP dissipated quickly into the membrane of the receiving cell (green arrow), and membrane-RFP was no longer observed 20 min after transfer (Fig. 3G).

**Figure 4:**
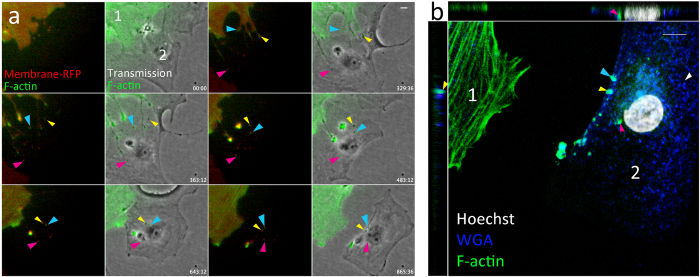
Exchange and internalization of membrane-RFP and F-actin-GFP through TNTs. (**a**) Different TNTs are formed between cell 1 and 2 (colored arrows indicate individual TNTs). These TNTs allow transfer of F-actin-GFP surrounded by membrane-RFP to the accepting cell. The particles are exchanged at different time points and remain visible until the end of the recording (865 min). (**b**) Subsequent confocal recording with orthogonal views of the accepting cell 2 shows internalized actin-GFP. Membrane is stained with WGA (blue). Colored arrows show internalized particles (arrow colors correspond to colors used in F). Scale bars represent 30 μm (D, E) or 10 μm (A–C, F–G).

We next investigated whether actin was present in the TNTs that were visible with brightfield and/or membrane-RFP, and found that both types of TNTs contained F-actin (Fig. 4A). Multiple TNTs were formed between cell 1 and cell 2 through cell dislodgement during this recording, and small particles labeled with membrane-RFP and actin-GFP transferred from cell 1 to cell 2 during the recording. After 865 min, the pericytes were fixed and stained with WGA to visualize the membrane in cell 2. Subsequent confocal recording shows that these actin/membrane particles were internalized into the accepting cell (cell 2; Fig. 4B).

### Quantification of length and number of tunneling nanotubes

**Figure 5:**
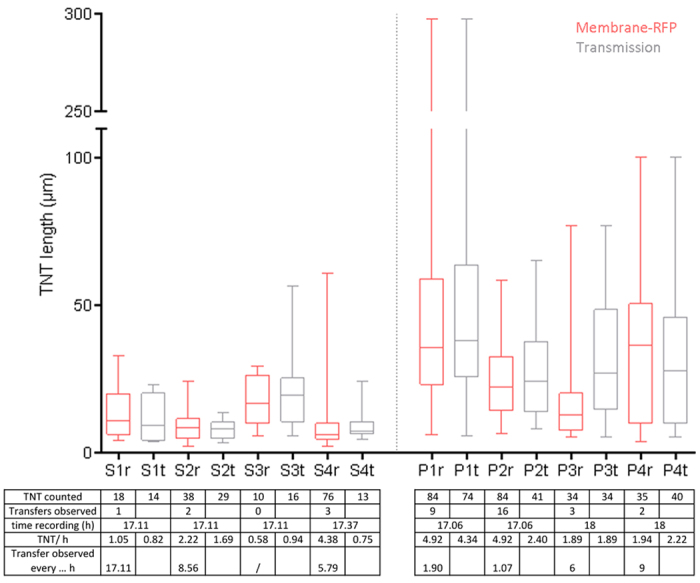
Boxplot representing lengths of TNTs as measured with brightfield (t; grey boxplots) or with membrane-RFP (r; red boxplots) in four SH-SY5Y cells (S1-S4) and four pericytes (P1-P4). The table below the boxplot shows the number of actual observed membrane-RFP TNT-mediated transfers between these cells during the same period.

Transfer of cargo is required for a process to be a genuine TNT. This is often omitted in TNT-related research, but is important as it distinguishes TNTs from other, similar-looking structures. We quantified the number of TNTs in SH-SY5Y cells and primary pericytes, and found a large variation in the number and length of TNTs. The difference in TNT number between cells was mostly attributable to the number of interacting cells and the motility of the individual cells (Movies 1 and 2). TNT numbers were obtained by quantifying the TNTs twice and by making a distinction between the TNTs that were visible with either membrane-RFP or with brightfield. For this, four different fluorescent cells were followed over time (17–18 h) and TNTs were counted at forty time points, each 25.5 to 27 min apart, on each cell. This method allowed us to avoid counting TNTs that remained visible in sequential frames more than once. However, even using this method, several TNTs remained visible over multiple time points. This was especially true for the TNTs formed between human pericytes. TNTs between pericytes were longer, with lengths up to 297 μm vs. 61 μm in SH-SY5Y cells (Fig. 5). This difference will in part be caused by the pericytes being larger in size than SH-SY5Y cells. More TNTs were counted with membrane-RFP than with brightfield, because finer TNTs could only be seen in the fluorescence.

For the four SH-SY5Y cells, we counted 10–76 TNTs per cell with membrane-RFP and 13–29 TNTs per cell with brightfield, but we only observed 0–3 membrane transfers per cell over 17 h (Fig. 5, table). For the primary pericytes there were 34–84 TNTs per cell with membrane-RFP, and 34–74 TNTs per cell with brightfield. Here we observed 2–16 membrane transfers per cell over 17–18 h. The transfer of intracellular α-syn or control mCherry was not quantified in these recordings; however, our live-cell imaging data showed that TNT-mediated exchange of fluorescent α-syn or control mCherry was only observed in a small subset of cells. In the cells with α-syn or control mCherry exchange through TNTs, only one α-syn exchange was observed throughout the entire recording (average 24 h). This observation indicated that intercellular transport occurs less frequently than membrane exchange.

### Characterization of TNT properties

**Figure 6:**
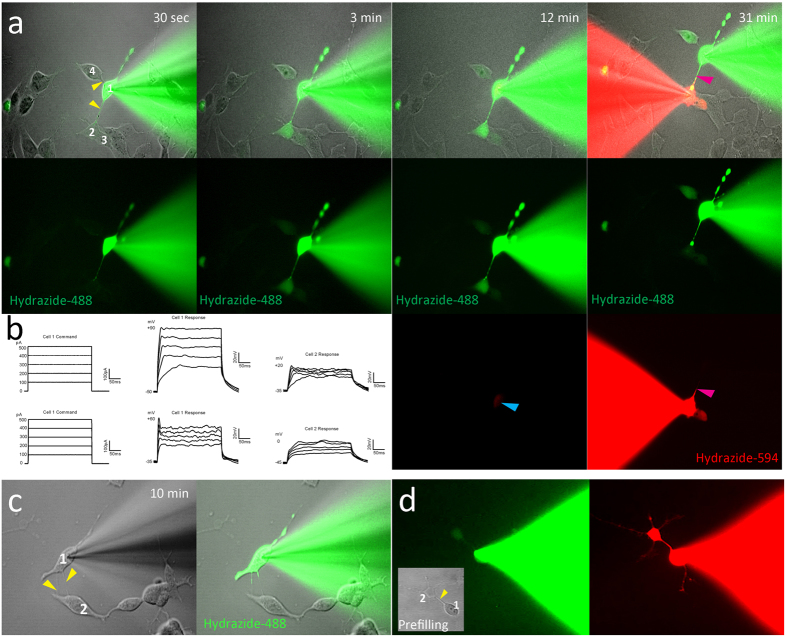
Characterization of tunneling nanotube (TNT) properties in SH-SY5Y cells. (**a**) Transfer occurred through TNTs (yellow arrows) within 30 sec of dye filling, and hydrazide-488 gradually increased in cells 2, 3, and 4. At 12 min, prior to filling cell 2 with hydrazide-594 a small amount of bleed through is present in the red channel (blue arrow). Dye filling of cell 2 with hydrazide-594 began at 22 min, and by 31 min, the TNT connecting cells 1 and 2 filled up with hydrazide-594 (pink arrow). (**b**) Electrical connectivity of SH-SY5Y cells 1 and 2 (from figure A) connected through a TNT 45 min after the dye filling of cell 1 began. (**c**) Closed TNT: no transfer of hydrazide-488 through the TNT after 10 min of dye filling cell 1. Yellow arrows indicate TNTs. (**d**) Open TNT: cell 1 was simultaneously filled with dextran (10 kDa)-488 and hydrazide-594. After 6 min both dextran (10 kDa)-488 and hydrazide-594 had transferred through the TNT.

To determine the electrical properties and type (i.e., if they were open or closed TNTs), cells with TNTs were patch clamped and injected with fluorescently tagged hydrazide. Some TNTs, but not all, allowed transfer of material between cells. Hydrazide-488 transferred through TNTs to connected cells within 30 sec (Fig. 6A) and could form networks of interconnected cells that allowed intercellular transport via TNTs (cell 1 fills up cells 2 and 4; cell 2 then fills up cell 3). Injecting hydrazide-594 in cell 2 indicated that TNTs allow bidirectional transport.

Electrical connectivity between a TNT-connected cell pair was indicated with a voltage response in cell 2 in response to current injection in cell 1. Electrical connectivity in the reverse direction (i.e. from cell 2 to cell 1) was also tested (Fig. 6B). We patched and injected a similar-looking cell with possible TNTs. However, these TNTs were closed, as they did not allow transfer of hydrazide-488 even after 55 min of dye filling (Fig. 6C). The right-hand panel (Fig. 6C) clearly shows the makeup of the closed TNT, with the left TNT filling up halfway and the right TNT filling up to one-third.

These results show that morphological characterization is not sufficient to identify TNTs, because although the TNTs look similar (Fig. 6A,C), they do not all allow transport. Different types of TNTs have been described previously: they can be open, allowing contact of cytoplasm; closed but regulated through gap junctions, allowing transport of small particles (<2 kDa); or closed TNTs that do not allow a continuous exchange^11^. To determine if the TNTs that allowed transport were gap-junction-regulated or open tubes, we simultaneously injected dextran-488 (10 kDa) and hydrazide-594 (758.79 Da). As gap junction transport is limited to transporting cargo up to 1–2 kDa, adding a larger sized complex allows discrimination between these two types of TNT^16^. Lower molecular weight hydrazide-594 transferred faster and further into the processes of cell 2, with transfer observed after 30 sec. Dextran-488 (10 kDa) became visible in cell 2 after 3 min and then filled up the cell body. Both dextran-488 and hydrazide-594 were transferred through the TNT, indicating that this TNT was an open tube (Fig. 6D).

## Discussion

Irrespective of the cause of PD (genetic or sporadic) there is an accumulation and spread of α-syn, which leads to the pathology of the disease. However, the role of non-neuronal cells such as pericytes is unclear. In this paper, we demonstrated for the first time that TNTs are involved in transporting α-syn between human non-neuronal cells and suggest that TNTs could play a role in the spread of α-syn, potentially in a prion-like way. Pericytes connect endothelial cells, astrocytes and neurons together. As such α-syn transfer through pericytes could provide an alternative non-neuronal transport mechanism. Together with the recent finding that TNTs allows α-syn transfer in primary mouse neurons^10^ it emphasizes the importance that TNT could play in the spread of α-syn in PD.

Transport through the TNT is rapid and transported particles can be observed in the neighboring cell within 30 sec. TNTs can transfer large and small particles, as well as cell membrane. TNTs are frequently formed *in vitro.* However, due to the lack of a specific TNT staining further work needs to be done to identify a unique marker^17^,^18^. *In vivo* reports of TNTs are still limited and the reason to downplay the results from *in vitro* culture experiments. However, Osswald *et al*., have described the function of TNT-like structures in brain tumors in mice. They clearly show their importance *in vivo* as cells connected through TNT-like structures were protected from cell death inflicted by radiotherapy^19^. Our research was performed on two cell types to derive more reliable conclusions. *In vitro* transfer studies in SH-SY5Y cells were confirmed in our primary pericytes cultures. *In situ* we found pericytes containing α-syn precipitates *and* TNT-like structures that are similar to TNTs observed in human pleural mesothelioma^17^.

Previously it has been shown that endocytosis^20^, the release of pathogenic α-syn from dying cells^21^, and exosome exchange^22^ are all involved in spreading α-syn between cells. Our findings suggest an additional mechanism explaining how α-syn pathology can spread throughout the brain. Both SH-SY5Y cells and human brain pericytes frequently form TNTs that function as a conduit for α-syn transfer between cells and long-distance electrical coupling. Transport was only observed in a small subset of cells. Although intercellular transport of α-syn is limited, transfer through TNTs could play an important role over an extended time period. We demonstrated that transported α-syn remains visible over extended time periods (>72 hours) indicating that some cells have difficulty clearing α-syn. Combining this with the decreased lysosomal function that occurs in PD, even infrequent TNT-mediated transport may be sufficient for defective α-syn to propagate within the brain^23^. Fluorescent α-syn, fluorescent control protein, and fluorescent membrane all used TNTs to transfer between cells, which suggests that this transport is part of a more general information exchange mechanism. It is not surprising that TNTs in SH-SY5Y cells and pericytes facilitate α-syn spreading between cells as TNTs are able to transfer a variety of material, such as RNA, organelles, vesicles, proteins, HIV, signaling molecules and prions^11^,^12^,^14^,^23^,^24^,^25^.

From the start, we used very strict criteria to define a TNT but only observing an actual transfer can unmistakably separate TNTs from similar-looking structures like filopodia and other neuronal processes such as dendrites and axons^10^. This aspect of TNT identification is frequently omitted because of the difficulty in seeing cargo being transported^26^,^27^. We could not visually differentiate open from closed TNTs in our studies, and only by injecting fluorescent dye into cells or observing transfer could this distinction be made. Our counting data of live cell imaging shows that the majority of TNTs did not result in a transfer. Previous studies might also have under-estimated the number of TNTs, as our experiments with BacMam-induced membrane-RFP have shown. We counted many more TNTs using membrane-RFP than with brightfield. The temporary increase in TNT numbers when cells go through mitosis might play an important role in α-syn transport, not necessarily between mature neurons, but because other replicating non-neuronal cell types (like the brain-derived pericytes used in this study) could play a role *in vivo* in the spread of α-syn.

Compared to the infrequent intercellular transport of α-syn, we detected a high number of membrane exchanges in SH-SY5Y cells and brain pericytes. The exchange of membrane through TNTs could play an important role during intercellular transport and the spread of α-syn. Several studies have shown the interaction of α-syn with the membrane, which alters the cellular function. Neurons overexpressing α-syn display increased permeability and α-syn oligomers could interfere with the normal functions of cellular membranes and form pore-like structures^28^. This could function as a self-enforcing mechanism enhancing membrane and α-syn spread. Furthermore α-syn mutants A53T and E46K exhibit increased membrane-binding affinity^29^. Although we did not see a difference between constructs in TNT mediated transfer, the close interaction of α-syn with the membrane might be sufficient for α-syn to be transported together with the membrane exchange we frequently observed.

In summary, TNTs function as a conduit for α-syn exchange in SH-SY5Y cells and non-neuronal pericytes. However, TNT-mediated transport is not restricted to α-syn or membrane exchange, but likely plays a more general role in cellular communication. This manuscript provides further evidence that a-syn spread can be attributed to TNTs and that pericytes, a key non-neuronal cell type in the blood brain barrier, could be involved in this process. As a-syn spread is seen as a contributor to PD pathology, elucidating mechanisms of this transfer may offer additional therapeutic targets to conventional PD therapy.

## Materials and Methods

### Brain tissue

Brains were acquired with informed consent from all families and this process was approved by the University of Auckland Human Participants Ethics Committee (2014/011654). All methods in this study were performed in accordance with the relevant guidelines and regulations. PD brain tissue was diagnosed by a neuropathologist. For the immunohistochemical studies, human brains were processed as previously described^30^. The cases used in this study were: PD63 (91 years, female, 5 h postmortem delay), PD65 (67 years, male, 2 h postmortem delay), PD67 (65 years, male, 17 h postmortem delay), and control middle temporal gyrus (MTG; 65 years, male, 8 h post mortem delay).

### Cell culture and transfection

Middle temporal gyrus (MTG) tissue from two PD human brains (PD63, PD65) were collected postmortem, as previously described^15^. In short, tissue containing the MTG was mechanically dissected and dissociated prior to being enzymatically digested in Hank’s Balanced Salt Solution (HBSS) containing 2.5 U/mL papain (Worthington) and 100 U/mL DNase 1 (Invitrogen) for 30 min at 37 °C with gentle rotation, which included a gentle trituration step at 15 min. Enzymatic digestion was halted by the addition of complete culture media; DMEM:F12 (Invitrogen) containing 10% fetal bovine serum (FBS; Gibco), 1% Penicillin/Streptomycin (Gibco), and GlutaMAX (Invitrogen). Cells were collected by centrifugation (170 *g *×* *10 min), resuspended in the complete culture media, and plated onto uncoated T75 culture flasks (Nunc). Cells were passaged up to passage 9 with early passages (2–3) containing microglia, astrocytes and pericytes, whilst latter passages contained only pericytes. Detailed characterization of both early and late passage cultures has been performed previously^15^,^31^. In general, these cultures contain 85% pericytes, ~10% CD45^+^ microglia, and ~5% GFAP^+^ astrocytes in the first two passages. Passage 5 and above cultures contained 100% pericytes. Under our *in vitro* conditions microglia and astrocytes do not proliferate and are diluted out in subsequent passages. To ensure there was no contamination from microglia in pericyte cultures only passages 5-9 were used. Late passage cultures showed positive immunocytochemical staining for the pericyte markers platelet derived growth factor receptor beta (PDGFRβ), alpha smooth muscle actin (αSMA), and neural/glial antigen 2 (NG2) as well as the fibroblast markers prolyl-4-hydroxylase (P4H) and fibronectin. There were no cells positive for the microglia marker CD45 or the astrocyte marker glial fibrillary acidic protein (GFAP) from passage 5 onwards^15^,^31^. Cells were incubated at 37 °C with 5% CO_2_ until seeded for experiments. SH-SY5Y cells (CRL-2266™) were obtained from ATCC. Both cell types were also grown in DMEM:F12 (Invitrogen) containing 10% FBS (Gibco) and 1% Penicillin/Streptomycin (Gibco).

Stable transfected SH-SY5Y cells were generated with Lipofectamine 3000 (Invitrogen) using the following constructs: α-syn-WT-EGFP and α-syn-A53T-EGFP (40822 and 40823, Addgene^32^), α-synuclein-mCherry/pcDNA3.1 (N-terminal tagged), and mCherry/pcDNA3.1^33^. All constructs were sequenced on arrival and western blots were performed on overexpressing cells. Pericytes were transiently transfected with Lipofectamine 3000 (Invitrogen). BacMam Plasma Membrane-RFP and actin-GFP (C10608 and C10506, Invitrogen) were used according to manufacturer instructions.

### Immunofluorescent labeling of paraffin sections

Immunohistochemistry was performed on 7 μm thick formalin-fixed, paraffin embedded samples and stained as described previously^34^ with primary antibodies raised against platelet derived growth factor receptor β (PDGFRβ), Abcam (ab32570, 1:200) and α-syn phospho, Abcam (ab184674, 1:3,000). Negative controls in which the primary antibody was omitted were included for each staining.

### Live cell recording, confocal imaging and scanning electron microscopy (SEM)

SH-SY5Y cells stably expressing α-syn-EGFP, α-syn-A53T-EGFP, α-syn-mCherry, or mCherry were used to study exchange through TNTs. Transient transfection of pericytes or SH-SY5Y cells with α-syn constructs or BacMam was performed directly in the petri dish. Images were captured over a 24–72 h period in a Nikon Biostation cell incubator (5% CO_2_, 37 °C). During imaging no readily identifiable adverse phototoxic effects were observed on the cells. All confocal recordings were imaged using an FV1000 confocal microscope (Olympus) with a 40x oil immersion lens (NA 1.00). SH-SY5Y cells for SEM were processed as described before^35^. Imaging occurred on an FEI Quanta 200 field emission Environmental SEM.

### Electrophysiology

For electrophysiology recordings SH-SY5Y cells, grown on poly-D-lysine coated coverslips, were transferred to a live cell recording chamber and visualized with DIC optics at 40x magnification on a Zeiss Axioscope upright microscope. All recordings were performed at room temperature in standard, carbogenated (95% O_2_, 5% CO_2_) artificial cerebrospinal fluid (in mM: 119 NaCl, 2.5 KCl, 1.3 MgSO, 4.7 H_2_O, 2.5 CaCl_2_, 1 NaH_2_PO4, 26 NaHCO_3_, 11 glucose; pH 7.2). Borosilicate glass recording microelectrodes were filled with internal solution (in mM: 120 K gluconate, 40 HEPES, 5 MgCl_2_, 2 NaATP, and 0.3 NaGTP; pH 7.2). Dual whole cell patch recordings were performed on potential TNT-connected cell pairs with patch pipettes of 6–8 MΩ. TNTs were visualized by filling SH-SY5Y cells through the patch pipette with 50 μM hydrazide-488 (570.48 Da) or hydrazide-594 (758.79 Da) and/or 250 μM dextran (10 kDa)-488 anionic and visualized with fluorescence. Prior to filling, the cells were imaged for any autofluorescence. To test for electrical connectivity between TNT-connected pairs of cells, one of the cells was held in current clamp mode and the membrane potential stepped for 300 ms from the resting membrane potential to depolarizing potentials ranging from 100-500 pA, in 100 pA increments. The second cell was also held in current clamp mode but without any current injection and the voltage response was recorded.

## Additional Information

**How to cite this article**: Dieriks, B. V. *et al*. α-synuclein transfer through tunneling nanotubes occurs in SH-SY5Y cells and primary brain pericytes from Parkinson’s disease patients. *Sci. Rep.* **7**, 42984; doi: 10.1038/srep42984 (2017).

**Publisher's note:** Springer Nature remains neutral with regard to jurisdictional claims in published maps and institutional affiliations.

## Supplementary Material

Supplementary Movies Legend

Supplementary Movie 1

Supplementary Movie 2
