# Supplementary material for: α-synuclein transfer through tunneling nanotubes occurs in SH-SY5Y cells and primary brain pericytes from Parkinson’s disease patients
Source: Sci Rep. 2017 Feb 23;7:42984. doi: 10.1038/srep42984 (PMC5322400; doi:10.1038/srep42984)
Supplement: Supplementary Movies Legend [file srep42984-s1.pdf]

Supplementary information

α-synuclein transfer through tunneling nanotubes occurs in SH-SY5Y cells and primary brain pericytes from Parkinson's disease patients

Birger Victor Dieriks[1,4], Thomas I-H. Park[2,4], Chantelle Fourie[3,4], Richard L.M. Faull[1,4], Mike Dragunow[2,4], Maurice A. Curtis[1,4]

[1]Department of Anatomy and Medical Imaging, [2]Department of Pharmacology, [3]Department of Physiology, [4]Centre for Brain Research, Faculty of Medical and Health Science, University of Auckland, Private Bag 92019, Auckland, New Zealand.

Supplementary movies

Movie 1 Formation of TNTs and transfer of α-syn A53T EGFP in SH-SY5Y cells. A TNT connecting an α-syn A53T EGFP expressing cell with a mcherry expressing cell is shown. The TNT is connected from the start of the recording. When the TNT retracts, three individual α-syn A53T EGFP particles are observed in the accepting mCherry cell (see zoom). These particles appear to merge into one larger particle and remain clearly visible until 615 min after which the α-syn A53T EGFP is no longer seen. Scale bar represents 10 µm.

Movie 2 Formation of TNTs and transfer of mCherry in SH-SY5Y cells through TNTs during mitosis. Combined recording of transmission and fluorescent mCherry with zoom of mCherry on the right hand side. At the start of the recording most TNTs are formed through connected cells migrating away from each other. As the recording continues and cells are further apart, TNTs are mostly formed through protrusion of one cell to the other. At 1052

min mitosis occurs in the bottom right mCherry cell, during which time multiple connections are formed with substrate and neighbouring cells. The TNT formed between the mCherry cell and the non transfected cell above allows for transfer of mCherry (see zoom on right hand side), which remains in the cell for over 6 h (500 min). Scale bar represents 10 µm.
